# Virtual Health Care for Community Management of Patients With COVID-19 in Australia: Observational Cohort Study

**DOI:** 10.2196/21064

**Published:** 2021-03-09

**Authors:** Owen Rhys Hutchings, Cassandra Dearing, Dianna Jagers, Miranda Jane Shaw, Freya Raffan, Aaron Jones, Richard Taggart, Tim Sinclair, Teresa Anderson, Angus Graham Ritchie

**Affiliations:** 1 Royal Prince Alfred Virtual Hospital Sydney Local Health District Camperdown Australia; 2 Sydney Local Health District Camperdown Australia; 3 Faculty of Medicine and Health University of Sydney Sydney Australia; 4 Menzies Centre for Health Policy University of Sydney Sydney Australia

**Keywords:** COVID-19, digital health, health, informatics, remote monitoring, telehealth, virtual health care

## Abstract

**Background:**

Australia has successfully controlled the COVID-19 pandemic. Similar to other high-income countries, Australia has extensively used telehealth services. Virtual health care, including telemedicine in combination with remote patient monitoring, has been implemented in certain settings as part of new models of care that are aimed at managing patients with COVID-19 outside the hospital setting.

**Objective:**

This study aimed to describe the implementation of and early experience with virtual health care for community management of patients with COVID-19.

**Methods:**

This observational cohort study was conducted with patients with COVID-19 who availed of a large Australian metropolitan health service with an established virtual health care program capable of monitoring patients remotely. We included patients with COVID-19 who received the health service, could self-isolate safely, did not require immediate admission to an in-patient setting, had no major active comorbid illness, and could be managed at home or at other suitable sites. Skin temperature, pulse rate, and blood oxygen saturation were remotely monitored. The primary outcome measures were care escalation rates, including emergency department presentation, and hospital admission.

**Results:**

During March 11-29, 2020, a total of 162 of 173 (93.6%) patients with COVID-19 (median age 38 years, range 11-79 years), who were diagnosed locally, were enrolled in the virtual health care program. For 62 of 162 (38.3%) patients discharged during this period, the median length of stay was 8 (range 1-17) days. The peak of 100 prevalent patients equated to approximately 25 patients per registered nurse per shift. Patients were contacted a median of 16 (range 1-30) times during this period. Video consultations (n=1902, 66.3%) comprised most of the patient contacts, and 132 (81.5%) patients were monitored remotely. Care escalation rates were low, with an ambulance attendance rate of 3% (n=5), emergency department attendance rate of 2.5% (n=4), and hospital admission rate of 1.9% (n=3). No deaths were recorded.

**Conclusions:**

Community-based virtual health care is safe for managing most patients with COVID-19 and can be rapidly implemented in an urban Australian setting for pandemic management. Health services implementing virtual health care should anticipate challenges associated with rapid technology deployments and provide adequate support to resolve them, including strategies to support the use of health information technologies among consumers.

## Introduction

Australia has been remarkably successful in controlling the COVID-19 pandemic, having reported some of the lowest numbers of cases and deaths among other high-income countries [1]. This success is attributed to a rapid nationwide coordinated public health response to implement strong control measures that include widespread testing and contact tracing, social distancing, prohibition of public gatherings, use of face masks, and restrictions on international and domestic travel, including a mandatory quarantine period for those arriving from other countries [2,3]. The Australian government has also implemented nationwide funding for telehealth services, which is now a permanent reform, leading to rapid adoption of telehealth services [4].

COVID-19 manifests relatively mildly in most cases; however, approximately 14% of infected individuals develop severe disease that requires hospitalization, and 5% require admission to an intensive care unit [2]. The vast majority of COVID-19 cases in Australia have been actively monitored and managed by the public health system, and this arrangement was sustainable owing to the small number of cases and effective contact tracing systems. However, it was initially speculated that the Australian health care system had inadequate acute care facilities and intensive care unit beds to manage the high expected case load, which led to the rapid exploration of new models of acute care [5,6].

Virtual health care (VHC) is a model of health service delivery, which substitutes in-person consultations with telephonic or video consultations and often includes asynchronous data collection from the patient via survey tools with or without real-time remote monitoring. VHC is emerging as a central strategy to manage large numbers of medical patients affected by the COVID-19 pandemic, as this can maximize the use of limited clinical resources, reduce pressure on acute care facilities, and reduce the risk of health care–associated infections [7]. Studies have reported high satisfaction rates for telehealth services among patients and clinicians, with comparable clinical and service outcomes for chronic diseases [8]. Smith et al [9] reported on telehealth deployments in other crises including hurricanes Harvey and Irma. VHC-based models of acute care for COVID-19 management in high-income countries are emerging [10-17]. However, few models that include continuous remote monitoring of clinical observations and extensive utilization of videoconferencing platforms have been reported.

This study describes the rapid deployment of a VHC model that includes remote monitoring of clinical observations and routine use of videoconferencing platforms within a large Australian metropolitan public health service for managing outpatients with COVID-19.

## Methods

### Setting

Sydney Local Health District (SLHD) is a large metropolitan public health service in New South Wales, Australia, encompassing 5 hospitals, 4 large community health centers, and 12,000 staff. SLHD is responsible for the health and well-being of approximately 700,000 individuals living within its geographic boundaries and approximately 1,000,000 individuals who travel to the city each day for work, study, and recreation [18].

SLHD has been implementing telehealth and VHC modalities for several years. On February 3, 2020, SLHD commenced operations of the Royal Prince Alfred Virtual Hospital (rpavirtual), Australia’s first metropolitan virtual hospital [19]. This service was established as a 12-month pilot program providing at-home and remote web-based nursing services. The initial patient cohorts included those seeking palliative care, adult patients with cystic fibrosis, and those at the risk of recurrent lower leg wounds. rpavirtual has a robust operational and clinical governing body including a general manager and a clinical director to oversee its operations, and it is embedded in the organizational structure of the health service, which includes a dedicated public health unit (PHU).

Colocated in the Royal Prince Alfred Hospital campus in Camperdown, New South Wales, rpavirtual is a 24/7 care center with technology-enabled multidisciplinary team rooms, handover areas, tracking boards, and several care pods. The care pods provide access to the electronic medical record (EMR) and shared care planning and remote monitoring tools. They are equipped with videoconferencing and telephone facilities and are staffed by nurses who can remotely monitor multiple patients simultaneously. The facility has medical staff on site and is under the supervision of a clinical director and director of nursing. A team of >100 community nurses are available to deliver in-home nursing care to complement VHC services.

On March 5, 2020, in response to the COVID-19 pandemic in Australia, rpavirtual began a rapid redesign of care systems to provide VHC to patients with COVID-19 managed in the community. The first patients were enrolled on March 11, 2020. This study describes the early experience with VHC use to manage patients with COVID-19 in the community up to and including March 29, 2020. The original patient cohorts continued receiving VHC and their outcomes are described separately.

The SLHD ethics review committee reviewed this study, and no ethical concerns were raised regarding the study or publication of the results.

### Population

Patients who attended COVID-19 testing clinics of the SLHD and tested positive were informed of their outcomes by the local PHU and referred to the rpavirtual care center. The care center conducted an initial telephonic clinical assessment to ascertain suitability for VHC. Inclusion criteria and relative exclusion criteria are listed in Textbox 1.

**Selection criteria of the Royal Prince Alfred Virtual Hospital for virtual health care for patients with COVID-19.**

**Inclusion criteria**

- The public health unit is satisfied that the patient can self-isolate safely and understands how to manage self-isolation.
- The clinical team is satisfied that it is clinically appropriate to manage the patient at home and a caregiver can safely provide care to the patient with appropriate personal protective equipment, or the patient can provide care to himself/herself, and a member of the clinical team carries out daily monitoring and follow-up evaluation.

**Relative exclusion criteria**

- Individuals over 65 years of age with significant comorbidity including, but not limited to, cancer, cardiovascular disease, diabetes, heart failure, immunosuppression, stroke, liver disease, renal disease, and lung disease.
- Individuals under 65 years of age with one or more of the following comorbidities: lung disease, cardiovascular disease, renal disease (including stage 5 chronic kidney disease or requiring renal replacement therapy including renal transplantation).
- Uncontrolled hypertension.
- Individuals at residential aged care facilities.
- Individuals aged <18 years.
- Pregnant women.

Patients matching one of the relative exclusion criteria were still accepted into VHC subject to further discussion with their treating physicians or an emergency medicine specialist. To be able to use the supplied technology and videoconferencing facilities, patients required access to a smartphone, tablet device, or personal computer with an internet connection and video capability.

Patients unable to self-isolate at home were offered alternative accommodation managed by the health service. Non–English-speaking patients were accepted with the support of interpreters. Patients deemed ineligible or unsuitable for VHC were required to be hospitalized. Multimedia Appendix 1 provides a schematic representation of the eligibility assessment process of rpavirtual for patients with COVID-19.

### Model of Care

The rpavirtual COVID-19 VHC model was based on early detection of deterioration and managed care escalation for deteriorating patients.

Vital signs, including respiratory rate, oxygen saturation, pulse rate, and temperature, were monitored at home. Blood pressure monitoring was not required. The care center contacted patients at pre-arranged intervals thrice a day, including a video consultation with the patient twice every 24 hours, thus facilitating further assessment of symptoms and signs of deterioration (Textbox 2) based on standard nursing assessment approaches [20,21].

**Telemedicine assessment items of the Royal Prince Alfred Virtual Hospital for patients with COVID-19. ^a^Clinical assessment was conducted through video consultations, direct observation, patient-reported observations, or with remote monitoring devices (for temperature only).**

**Clinical progress**

- Do you feel feverish or have chills?
- Do you have a cough?
- Do you feel short of breath or find it difficult to talk?
- Do you have any other symptoms?

**Psychological screening**

- How are you managing your home isolation?
- How are you coping generally?

**Clinical assessment^a^**

- Airway
- Breathing: respiratory distress, respiratory rate, oxygen saturation, and other respiratory symptoms
- Cardiovascular system: appearance and heart rate
- Disability: alertness, cognition, mental state, and mobility
- Exposure (temperature): temperature
- Fluid balance: fluid and food intake and gastrointestinal symptoms and losses
- Glycemic control (if diabetic): blood glucose levels

Vital signs were recorded electronically in the EMR and tracked against a standardized early warning system known as “Between The Flags,” using the standard criteria for observing adults in the general population (Multimedia Appendix 2) [22,23].

Clinical escalation of deteriorating patients was carried out through managed transfer to the local emergency department in an ambulance if clinically indicated. The ambulance service was notified of the patient’s infectious status and advised to contact the receiving emergency department prior to arrival. In the event of escalation, the care center also notified the hospital executive and the local PHU.

An escalation communication pathway was activated if a patient could not be contacted by the care center. If a patient could not be contacted after one telephone call, a text message was sent. If the patient did not revert within 1 hour, a second text message was sent, which expressed concern for their welfare. If the patient did not respond to a third telephone call, the New South Wales Police was contacted to conduct a welfare check.

Medical officers at rpavirtual were consulted through referral from the care center staff to discuss patient deterioration, escalation decision-making, medical certification, and prescribing medications, although medication management was not a component of the model of care.

Discharge from VHC was managed in accordance with the following “release from isolation” criteria [24]: the individual has been afebrile for the previous 48 hours, the acute illness resolved in the previous 24 hours, at least 7 days have elapsed since the onset of the acute illness, and a negative result was obtained on RT–PCR with at least two consecutive respiratory specimens collected 24 hours apart after the acute illness has resolved.

Patients meeting the first three criteria were referred to a COVID-19 testing clinic for repeat testing. On obtaining negative results, the patient was discharged from VHC and referred to their general practitioner for ongoing care.

### Patient Experience

The use of technology by patients and their caregivers is central to care delivery by rpavirtual. Once enrolled in VHC, new patients were provided with a welcome pack containing the following items: a welcome letter and videoconferencing instructions, pulse oximeter and instructions for its use, a temperature monitoring device and instructions for its use, New South Wales Government COVID-19 factsheets, and a patient responsibilities pamphlet.

Personal protective equipment (PPE) was provided immediately after COVID-19 testing and then resupplied with the welcome pack.

The welcome pack was delivered by a “Flying Squad” of health informatics staff wearing appropriate PPE upon completion of a home visit risk assessment. The rpavirtual Flying Squad instructed patients to wear PPE when answering the door and notified patients when they were 5 minutes away from their homes.

Patients interacted with the rpavirtual team through video consultations and a tollfree telephone service that operated 24/7. Calls were scheduled with the patient to collect their self-observations. Patients were advised to call the rpavirtual call center or an ambulance if they experienced deterioration.

### Remote Monitoring Technology

Remote monitoring devices were evaluated to collect patient-generated health data from patients with COVID-19 in accordance with defined criteria (Textbox 3).

**Criteria of the Royal Prince Alfred Virtual Hospital for remote patient monitoring technology (in random order).**

- Capable of measuring the desired clinical parameters (ie, heart rate, oxygen saturation, temperature, and respiratory rate)
- Capable of remote data collection and transmission (or able to be read by the patient)
- Usability for the medical team at Royal Prince Alfred Virtual Hospital
- Usability for patients with COVID-19 admitted to the Royal Prince Alfred Virtual Hospital
- Training and support requirements for the health informatics staff of Sydney Local Health District
- Listed on the Australian Register of Therapeutic Goods of the Therapeutic Goods Administration (Department of Health, Australian Government) or able to be fast-tracked (eg, Conformité Européenne– or US Food and Drug Administration–marked)
- Supply chain availability to meet the scale, speed, and demand of the COVID-19 response
- Cost
- Compliance with cyber security, data security, and privacy and infection control requirements

**Figure 1:**
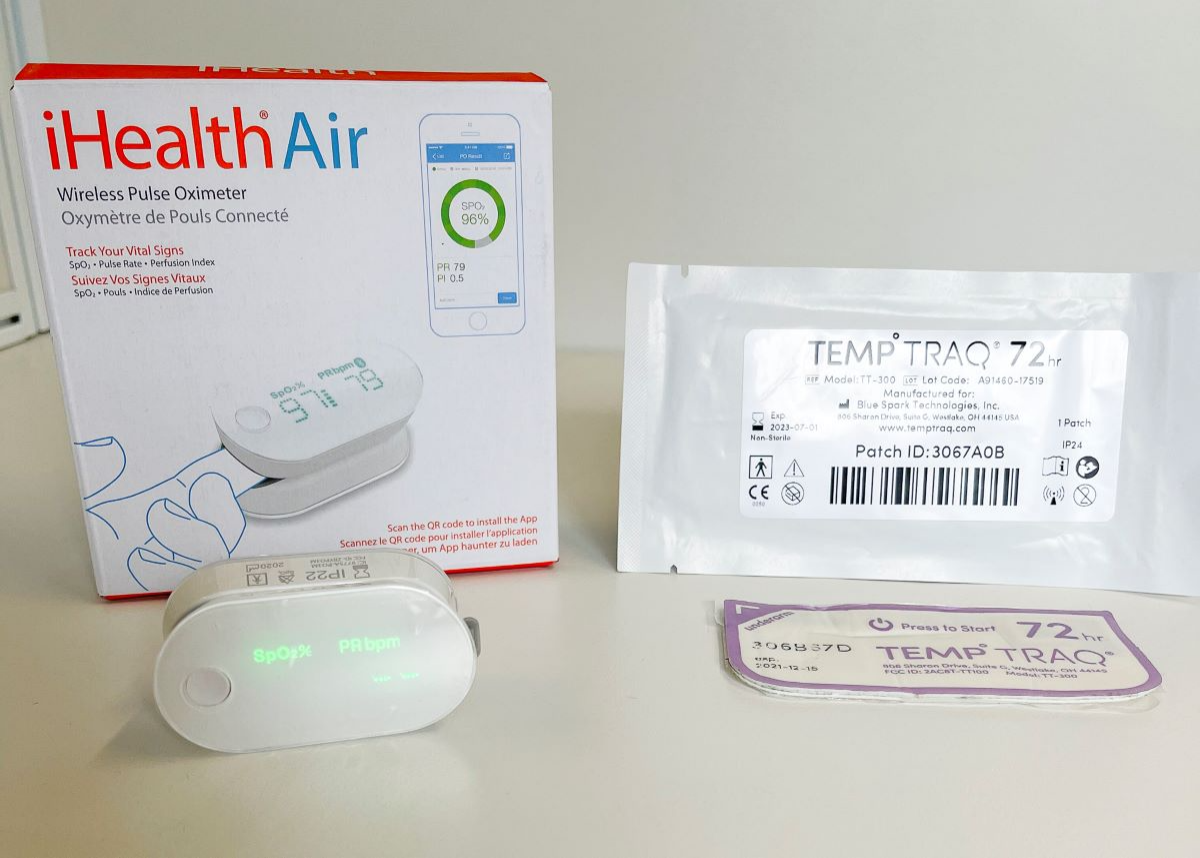
Remote monitoring equipment provided to patients for measurement of pulse, blood oxygen saturation, and temperature.

No suitable single device that met all vital sign monitoring requirements in accordance with the aforementioned criteria was identified. Two devices were selected for use in the COVID-19 program: a pulse oximeter and temperature patch (Figure 1). No reliable device for measuring the respiratory rate was identified; respiratory rate was measured through videoconference consultations.

A wireless pulse oximeter (iHealth Air pulse oximeter PO3M, iHealth Labs, Inc.) facilitated peripheral oxygen saturation and pulse rate measurements. Pulse oximeters were only used by one patient; they were not reused as they could not be adequately disinfected. A single-use, wearable temperature monitor (Temp°Traq Clinical, Blue Spark Technologies, Inc.) was self-applied in the axilla and facilitated continuous temperature monitoring. The device fed data continuously into a web-based dashboard, providing the care center with an overview of all patients. Each patch lasted 72 hours, and each patient was provided with 3 patches to cover the first 9-11 days of isolation.

Both devices had Bluetooth connectivity, but only the temperature monitor required connection with a compatible Apple or Android smartphone to be read; readings from the pulse oximeter were read directly from the device and reported through video consultations. Both devices were approved by the US Food and Drug Administration and Conformité Européenne–marked and either registered with the Therapeutic Goods Administration (Department of Health, Australian Government) or able to be fast-tracked but had only limited prior evaluation in the clinical setting [25,26].

**Figure 2:**
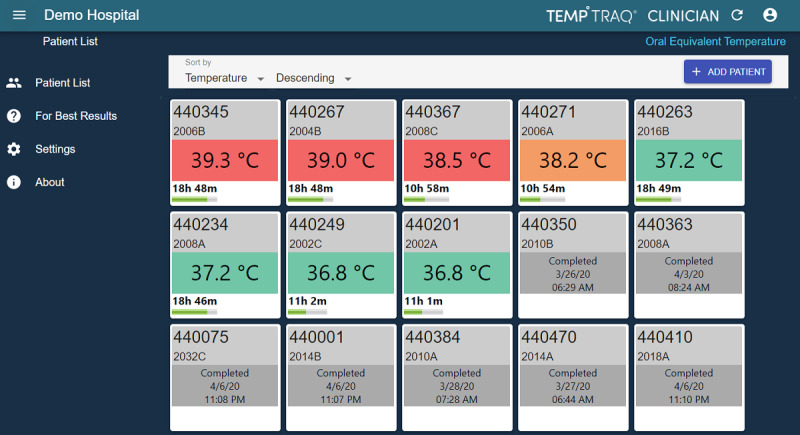
Screenshot of the temperature monitoring portal. This figure is from a demonstration system, and the temperature color indicators shown here are configured differently from the version in use: ≥38.0⁰C=red, 37.5⁰C-37.9⁰C=orange, 36.0⁰C-37.4⁰C=green, <36.0⁰C=blue. Expired or nonfunctioning patches are indicated in grey.

The Flying Squad prepared devices for delivery, including precharging of the pulse oximeters and registering of all 3 temperature monitoring patches against the specific patient in the monitoring portal. After delivery, they telephonically contacted the patient to help them download and set up the required mobile app, allowing temperature readings to be fed continuously into a web-based dashboard. The care center had access to an overview of all prevalent monitored patients with color-coded parameters to identify patients with an abnormal temperature or patches that did not function normally (Figure 2).

Patient-reported observations were manually documented in the EMR (Cerner Millennium, Cerner Corp) in purpose-built sections to allow them to be differentiated from other sources of vital signs.

### Video Consultations

Video consultations were carried out to visually assess the patient, confirm vital signs recorded by wearable devices, and to estimate the respiratory rate.

The rpavirtual care pods were fitted with high-definition webcams and Bluetooth-enabled headsets to allow the patient to see and hear the nurse. The care pods were configured to facilitate privacy during conversations with the patients. Pull-up backgrounds and feature walls were present behind each nurse to minimize distractions on video.

A commercial web-based video conferencing solution Pexip (Pexip AS) was used for remote video consultations. This platform functioned on both desktop and mobile devices and was endorsed by eHealth NSW, the state government agency overseeing the use of technology in health care. Patients called in to video consultations using their own computer or mobile device.

## Results

In March 11-29, 2020, 5821 individuals were tested for COVID-19 at SLHD COVID-19 testing clinics, and 173 individuals tested positive. Of them, 162 (93.6%) were enrolled in the rpavirtual program, and the remaining individuals were admitted to hospital or referred to another health service. The median age of the admitted patients was 38 (range 11-79) years; 3 patients aged <18 years and 2 pregnant women were admitted to VHC.

The median number of admissions per day was 6 (range 1-30). A total of 62 (38.3%) patients were discharged during this period, with a median length of stay of 8 (range 1-17) days. At the end of this period, 100 prevalent patients received VHC.

The care center commenced operations with 4 full-time–equivalent registered nurses and gradually increased to 9.5 full-time–equivalent at the end of the period, which equated to a ratio of approximately 25 patients per registered nurse per shift.

Patients were contacted 2865 times, with a median of 16 (range 1-50) contacts per patient. Video consultations (n=1902, 66.3%) comprised the majority of patient contact, and telephonic consultations (n=688, 24.0%) accounted for the remainder of patient contact. The ratio of telephonic-to-video consultations was 1:2.8. The median duration of each contact was 8.5 (IQR 5-15) minutes for telephonic consultations and 15 (IQR 13-15) minutes for video consultations.

During March 18-29, 2020, the rpavirtual Flying Squad delivered welcome packs with remote monitoring equipment to 132 of 162 (81.5%) patients.

Ambulances were called to attend to 5 patients, and 4 patients were transferred to the emergency department for assessment. In total, 3 patients were subsequently admitted, and 1 was discharged to continue with VHC at home. No deaths or police welfare checks were recorded. However, one individual was subject to a public health order for failing to adhere to self-isolation requirements. Detailed patient outcomes will be reported separately.

## Discussion

### Principal Findings

This study describes the rapid implementation of a model of care and technology to deliver VHC for community management of patients with COVID-19. We found that by excluding high-risk patients with COVID-19, we could include most individuals testing positive upon local diagnosis. Through remote clinical appraisal, supported by remote monitoring of clinical observations, we observed low rates of deterioration, with few patients requiring clinical escalation and no patient deaths. The program also enrolled pregnant women and pediatric patients, expanding the range of patients who may be suitable for VHC for COVID-19 as an alternative to hospital in-patient admission.

A range of technical and operational issues were expected with the rapid implementation of video consultations [8]. Our model of care required 3 patient contacts per day, with at least 2 contacts on video, to ensure adequate surveillance of signs of deterioration and to measure the respiratory rate directly. Initially, patients were scheduled for 10-minute video and telehealth consultations. On encountering a problem with video consultation, which the staff could not rapidly resolve, a telephonic consultation was carried out instead. This is an example of workaround—a common phenomenon when using health information technology that is driven by the need to resolve conflicting goals in a timely manner [27]. In this case, the workaround was driven by the need to adhere to the schedule of appointments rather than delaying to resolve the issue. Improving the user experience for staff and patients required both technical and workflow changes, and fixed appointment slots were abandoned. The optimal approach to training and support in VHC to avoid workarounds, particularly with a rapid increase in staff redeployed from other areas of the business (in the context of high service demands) requires further consideration.

The need for enhanced support for patients to use health information technologies was identified early in the implementation. The model of care relied on the ability of patients or their caregivers to use medical technology (pulse oximeter and temperature patch) and digital health applications (for temperature monitoring and video consultations). The use of health information technology by consumers, known as consumer health informatics, has a strong focus on usability and accessibility [28,29]. We immediately realized that patients enrolled in VHC could not easily download the temperature monitoring application and connect it with the patches with the instructions provided in the welcome pack alone. The welcome packs delivered by the Flying Squad, a multidisciplinary health informatics team, were ideally suited for providing additional support. A process was established to contact patients shortly after delivery of the welcome pack and support them in achieving the goal of transmitting temperature readings to the cloud-based monitoring panel. If consumer health technology is to be relied on for health care delivery, health services will require strategies to support it. While this has been explored in the chronic disease setting, in which patients can avail of in-person health services, this is not feasible during an infectious disease pandemic [30]. Managed mobile health care platforms, in which patients are provided with a tablet computer with all relevant applications pre-installed and connected to relevant peripherals, may simplify and improve the patient experience, and rpavirtual has used this approach with other patient cohorts. The role of health informaticians in supporting consumer health informatics has received limited attention and warrants further exploration.

A uniform model of care for all patients, regardless of care needs or the risk of deterioration, may not be appropriate or necessary. Risk stratification upon admission with enhanced monitoring of patients at a higher risk of deterioration was considered, although at the time, limited empirical evidence was available to guide that strategy [31]. Pulse oximetry appears to be a useful tool for risk stratification. Patients with COVID-19 experience deterioration typically on day 7 of symptom onset, which was the median period of hospitalization for patients who developed an associated pneumonia [32]. Patient-reported pulse oximetry measurements are effective for detecting silent hypoxia and predicting hospitalization; this supports the use of this technology for patient monitoring and risk stratification [16]. In addition to the primary management of COVID-19, pulse oximetry has effectively facilitated decisions on the early discharge of patients from hospital for a cohort of patients with severe COVID-19, with particular benefits among patients with a persistent need for oxygen therapy [13]. A risk-stratified care pathway has now been developed and is being used to guide ongoing patient management (Multimedia Appendix 3).

Rapid changes to the EMR are required to support VHC for patients with COVID-19. EMRs are potentially useful tools for rapid deployment of standardized processes, including responses to the COVID-19 pandemic [33]. To support the redesign of rpavirtual to provide care to patients with COVID-19, new locations and patient lists were generated in the EMR. Access to the electronic Between the Flags record for detecting signs of deterioration, previously only used in the in-patient setting, was extended to all care center staff for community use. Clinical documentation templates, simple reports, and modifications to the results flowsheet to facilitate recording and clinical review of patient-reported and remotely monitored clinical observations were designed and implemented. However, communication systems were managed separately from the EMR, with separate systems used for text messaging, telephone calls, and videoconferencing. A patient portal would have aided communication and recording of patient-reported measures, as previously reported [14,34]. A comprehensive and integrated suite of digital health care tools would reduce fragmentation of information systems and workflows and improve service delivery by automating manual processes.

Mental health and well-being issues require further consideration in the provision of VHC to patients with COVID-19 in Australia owing to extensive quarantine and self-isolation periods. Such measures for controlling infectious diseases have been associated with negative psychological effects including posttraumatic stress symptoms, confusion, and anger, with the potential to be long-lasting [35]. Two questions directed toward psychological assessment were included in the model to help recognize mental health concerns. The psychological impact of self-isolation in one’s own home may be different from that of individuals placed in mandatory quarantine in hotels after arriving from other countries, which has been a key strategy in Australia [3]. This issue requires further study with consideration to provide access to social workers, psychologists, and psychiatrists through VHC. The key learnings from our experience are summarized in Textbox 4.

**Key learnings from the use of virtual health care for acute management of patients with COVID-19 in Australia.**

- The acute nature of COVID-19 and the potential for rapid patient deterioration required 24/7 operations.
- A ratio of approximately 1 nurse per 25 patients per shift was required to support a model of care with high extensive of videoconferencing and continuous monitoring of patient observations with 24/7 operations; however, this could be reduced during low-activity periods such as during nighttime when patient contact was not scheduled.
- There was a high administrative burden in managing communication and interactions with enrolled patients when virtual health care is used without a digital patient engagement platform, such as a patient portal or other customer relationship management system with an integrated communications suite.
- Pulse oximetry appears to be an important tool in virtual care models for COVID-19 management for risk stratification and detection of deterioration and is feasible to use in the community. Readings that can be directly obtained from the device are useful.
- Consumers should be provided education, training, and support to use health technology if it is to be relied on for monitoring and management in virtual care settings.
- Social isolation measures introduce mental health risks that need to be considered within models of care, including screening tools and virtual access to appropriate services.

### Comparison With Previous Studies

The use of VHC for managing outpatients with COVID-19 has been widely reported with small cohorts, at single centers, or in health systems. A consistent finding is that VHC is safe and effective for hospital avoidance, with low rates of clinical care escalation to the emergency department or other in-person observations. However, there is significant heterogeneity in the models of care and technology models, with few reports on programs that highly utilize video consultations combined with remote patient monitoring of clinical observations, including pulse oximetry and temperature, along with 24/7 support.

The only other study from Australia, which described the generation and use of a virtual ward within an existing hospital, involved the referral of patients by the local public health service, and patients were contacted once or twice a day only by telephone [17].

Studies on the use of VHC for COVID-19 management in the United States have reported the extensive use of patient portals, patient-reported symptoms and observations, and the use of telephonic consultations, with a reduced use of remote patient monitoring systems and videoconferencing. One large health system used symptom questionnaires on a daily basis to stratify and prioritize enrolled patients with suspected or confirmed COVID-19 for telephonic consultations. This system reported low rates of escalation to the emergency department; however, there was no access to remote monitoring or video consultations. With increasing experience, a triage system was adopted to exclude low-risk patients owing to the low incidence of deterioration [14]. Another institution reported the need to expand their workforce to provide a 24-hour service because they found that the enrolled patients were sending text messages to their remote patient monitoring app after the service was discontinued each day at 5 PM [12].

Expansion of a model of VHC to the in-patient setting, based on continuous video observation, has also been described. Patients admitted for COVID-19 management were placed in negative pressure rooms equipped with video monitoring systems to facilitate 2-way video and audio communication with ward health care staff [34]. This reduced the exposure of health care workers to COVID-19 and the consumption of PPE.

### Limitations

This study has several limitations. First, this study has a small cohort, relative to other studies, and describes short-term experiences. Second, the technology used in this study was selected on the basis of pragmatic considerations, limiting opportunity for more thorough evaluation prior to use in practice. Finally, this study describes early results from a single health system in Australia and may not be generalizable to other locations. Australia has seen relatively smaller COVID-19 caseloads than other high-income countries, implying that the demand for health services did not exceed their current capacity to the same extent as that in other countries. Significant heterogeneity has been in described virtual health models of care for COVID-19, which are strongly influenced by context, pragmatism, and local constraints. Even so, sharing our experience may help inform others as they continue dealing with the pandemic.

### Conclusions

In summary, community-based VHC is a feasible and safe approach for managing less severe cases of COVID-19 and can be rapidly implemented in the Australian context for pandemic management with strong operational and clinical governance, including integration with clinical specialists. Health services implementing VHC should anticipate challenges with rapid technology implementations and provide adequate support to resolve them, including strategies to support consumer use of health information technologies.

## Appendix

Multimedia Appendix 1 Care centre eligibility criteria decision tree for COVID-19 management by rpavirtual.

Multimedia Appendix 2 Printout of 48h view of Standard Adult General Observation Chart with recorded clinical observations as shown in the electronic medical record.

Multimedia Appendix 3 rpavirtual COVID-19 risk stratification and clinical pathways.

## Abbreviations

EMR: electronic medical record
PPE: personal protective equipment
rpavirtual: Royal Prince Alfred Virtual Hospital
SLHD: Sydney Local Health District
VHC: virtual health care

## References

[1] Australia. World Health Organization.

[2] Chang SL, Harding N, Zachreson C, Cliff OM, Prokopenko M (2020). Modelling transmission and control of the COVID-19 pandemic in Australia. Nat Commun.

[3] Fotheringham P, Anderson T, Shaw M, Jewitt J, Storey H, Hutchings O, Cartwright J, Gupta L (2021). Control of COVID-19 in Australia through quarantine: the role of special health accommodation (SHA) in New South Wales, Australia. BMC Public Health.

[4] Thomas EE, Haydon HM, Mehrotra A, Caffery LJ, Snoswell CL, Banbury A, Smith AC (2020). Building on the momentum: Sustaining telehealth beyond COVID-19. J Telemed Telecare.

[5] Fox G, Trauer J, McBryde E (2020). Modelling the impact of COVID-19 on intensive care services in New South Wales. Med J Aust.

[6] Litton E, Bucci T, Chavan S, Ho Y, Holley A, Howard G, Huckson S, Kwong P, Millar J, Nguyen N, Secombe P, Ziegenfuss M, Pilcher D (2020). Surge capacity of intensive care units in case of acute increase in demand caused by COVID-19 in Australia. Med J Aust.

[7] Hollander JE, Carr BG (2020). Virtually Perfect? Telemedicine for Covid-19. N Engl J Med.

[8] Greenhalgh T, Wherton J, Shaw S, Morrison C (2020). Video consultations for covid-19. BMJ.

[9] Smith AC, Thomas E, Snoswell CL, Haydon H, Mehrotra A, Clemensen J, Caffery LJ (2020). Telehealth for global emergencies: Implications for coronavirus disease 2019 (COVID-19). J Telemed Telecare.

[10] Thornton J (2020). The "virtual wards" supporting patients with covid-19 in the community. BMJ.

[11] Torjesen I (2020). Covid-19: Patients to use pulse oximetry at home to spot deterioration. BMJ.

[12] Annis T, Pleasants S, Hultman G, Lindemann E, Thompson J, Billecke S, Badlani Sameer, Melton Genevieve B (2020). Rapid implementation of a COVID-19 remote patient monitoring program. J Am Med Inform Assoc.

[13] Grutters L, Majoor K, Mattern E, Hardeman J, van Swol C F P, Vorselaars A (2020). Home telemonitoring makes early hospital discharge of COVID-19 patients possible. J Am Med Inform Assoc.

[14] Kricke G, Roemer P, Barnard C, Peipert J, Henschen B, Bierman J (2020). Rapid Implementation of an Outpatient Covid-19 Monitoring Program. NEJM Catalyst Innovations in Care Delivery.

[15] Lam PW, Sehgal P, Andany N, Mubareka S, Simor AE, Ozaldin O, Leis JA, Daneman N, Chan AK (2020). A virtual care program for outpatients diagnosed with COVID-19: a feasibility study. CMAJ Open.

[16] Shah S, Majmudar K, Stein A, Gupta N, Suppes S, Karamanis M, Capannari J, Sethi S, Patte C (2020). Novel Use of Home Pulse Oximetry Monitoring in COVID-19 Patients Discharged From the Emergency Department Identifies Need for Hospitalization. Acad Emerg Med.

[17] Ferry OR, Moloney EC, Spratt OT, Whiting GFM, Bennett CJ (2021). A Virtual Ward Model of Care for Patients With COVID-19: Retrospective Single-Center Clinical Study. J Med Internet Res.

[18] (2019). Year in Review 2018-19. Sydney Local Health District.

[19] McDonald K (2020). Sydney LHD trialling virtual hospital for community care from RPA. Pulse+IT.

[20] Cathala X, Moorley C (2020). Performing an A-G patient assessment: a practical step-by-step guide. Nursing Times.

[21] Smith D, Bowden T (2017). Using the ABCDE approach to assess the deteriorating patient. Nursing Standard.

[22] Between the Flags 2020. New South Wales Government: Clinical Excellence Commission.

[23] Pain C, Green M, Duff C, Hyland D, Pantle A, Fitzpatrick K, Hughes C (2017). Between the flags: implementing a safety-net system at scale to recognise and manage deteriorating patients in the New South Wales Public Health System. Int J Qual Health Care.

[24] Coronavirus Disease 2019 (COVID-19). Australian Government: Department of Health.

[25] Moreno-Alsasua L, Garcia-Zapirain B, David Rodrigo-Carbonero J, Ruiz IO, Hamrioui S, de la Torre Díez Isabel (2017). Primary Prevention of Asymptomatic Cardiovascular Disease Using Physiological Sensors Connected to an iOS App. J Med Syst.

[26] Sampson M, Hickey V, Huber J, Alonso PB, Davies SM, Dandoy CE (2019). Feasibility of continuous temperature monitoring in pediatric immunocompromised patients: A pilot study. Pediatr Blood Cancer.

[27] Patterson ES (2018). Workarounds to Intended Use of Health Information Technology: A Narrative Review of the Human Factors Engineering Literature. Hum Factors.

[28] Eysenbach G (2000). Consumer health informatics. BMJ.

[29] Goldberg L, Lide B, Lowry S, Massett HA, O'Connell T, Preece J, Quesenbery W, Shneiderman B (2011). Usability and accessibility in consumer health informatics current trends and future challenges. Am J Prev Med.

[30] Milani RV, Bober RM, Lavie CJ (2016). The Role of Technology in Chronic Disease Care. Prog Cardiovasc Dis.

[31] Gong J, Ou J, Qiu X, Jie Y, Chen Y, Yuan L, Cao Jing, Tan Mingkai, Xu Wenxiong, Zheng Fang, Shi Yaling, Hu Bo (2020). A Tool for Early Prediction of Severe Coronavirus Disease 2019 (COVID-19): A Multicenter Study Using the Risk Nomogram in Wuhan and Guangdong, China. Clin Infect Dis.

[32] Wang D, Hu B, Hu C, Zhu F, Liu X, Zhang J, Wang B, Xiang H, Cheng Z, Xiong Y, Zhao Y, Li Y, Wang X, Peng Z (2020). Clinical Characteristics of 138 Hospitalized Patients With 2019 Novel Coronavirus-Infected Pneumonia in Wuhan, China. JAMA.

[33] Reeves J, Hollandsworth H, Torriani F, Taplitz R, Abeles S, Tai-Seale M, Millen Marlene, Clay Brian J, Longhurst Christopher A (2020). Rapid response to COVID-19: health informatics support for outbreak management in an academic health system. J Am Med Inform Assoc.

[34] Ford D, Harvey J, McElligott J, King K, Simpson K, Valenta S, Warr Emily H, Walsh Tasia, Debenham Ellen, Teasdale Carla, Meystre Stephane, Obeid Jihad S, Metts Christopher, Lenert Leslie A (2020). Leveraging health system telehealth and informatics infrastructure to create a continuum of services for COVID-19 screening, testing, and treatment. J Am Med Inform Assoc.

[35] Brooks SK, Webster RK, Smith LE, Woodland L, Wessely S, Greenberg N, Rubin GJ (2020). The psychological impact of quarantine and how to reduce it: rapid review of the evidence. Lancet.

