# Supplementary figures and images for: Virtual Health Care for Community Management of Patients With COVID-19 in Australia: Observational Cohort Study
Source: J Med Internet Res. 2021 Mar 9;23(3):e21064. doi: 10.2196/21064 (PMC7945978; doi:10.2196/21064)

# RPAVIRTUAL CARE CENTRE ELIGIBILITY DECISION TREE

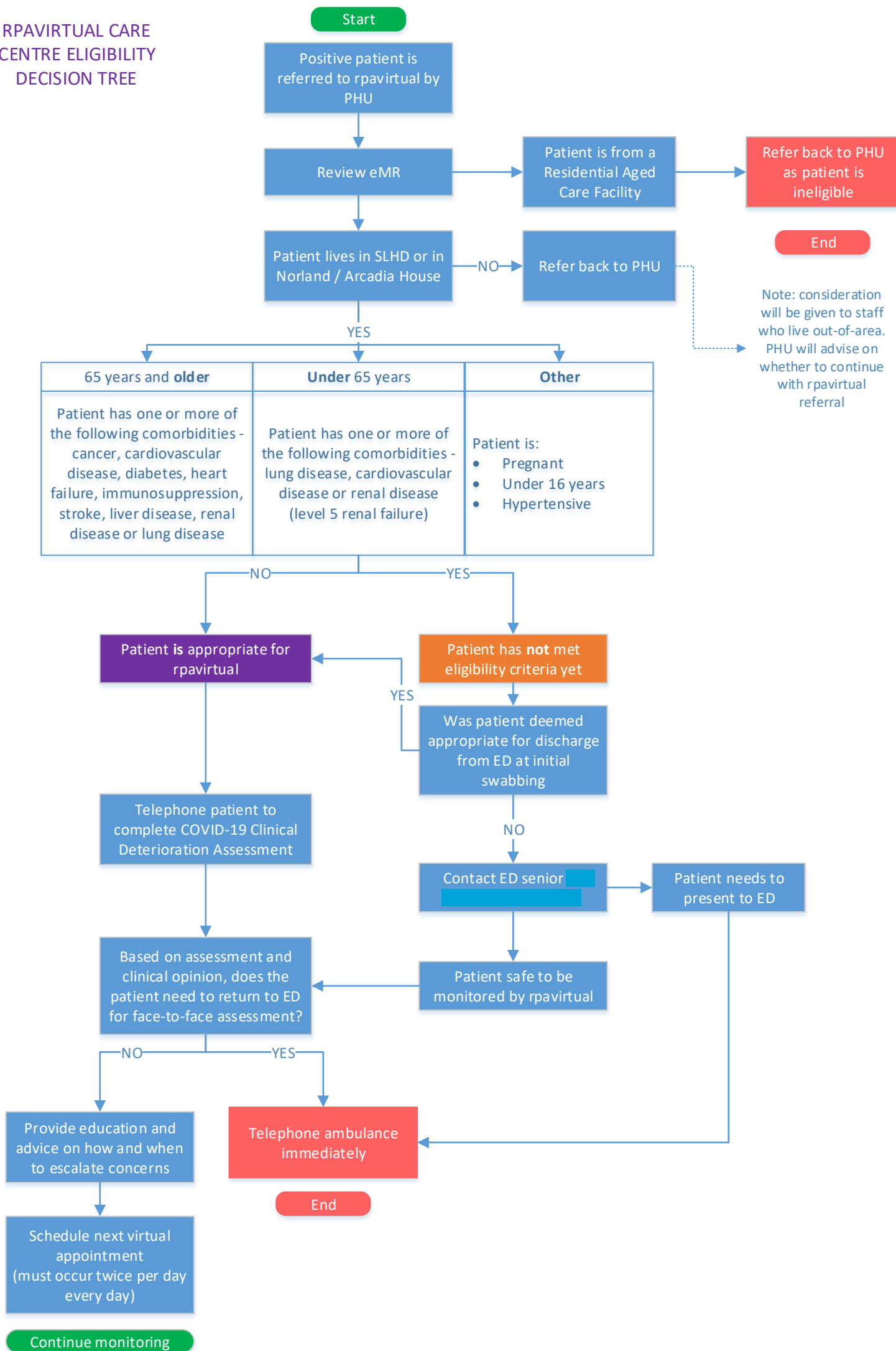

Supplement: Multimedia Appendix 1 [file jmir_v23i3e21064_app1.pdf]
