# Supplementary material for: Virtual Health Care for Community Management of Patients With COVID-19 in Australia: Observational Cohort Study
Source: J Med Internet Res. 2021 Mar 9;23(3):e21064. doi: 10.2196/21064 (PMC7945978; doi:10.2196/21064)
Supplement: Multimedia Appendix 2 [file jmir_v23i3e21064_app2.pdf]

REFERENCE ONLY [redacted]

SAGO Adult

Facility: [redacted]

[redacted]

MRN: [redacted] | [redacted]

Diagnosis: Visit Reason: COVID-19 Care and Monitoring Location: [redacted] DOB: [redacted] | [redacted]

Printout excludes Vital Signs with no results in this period.

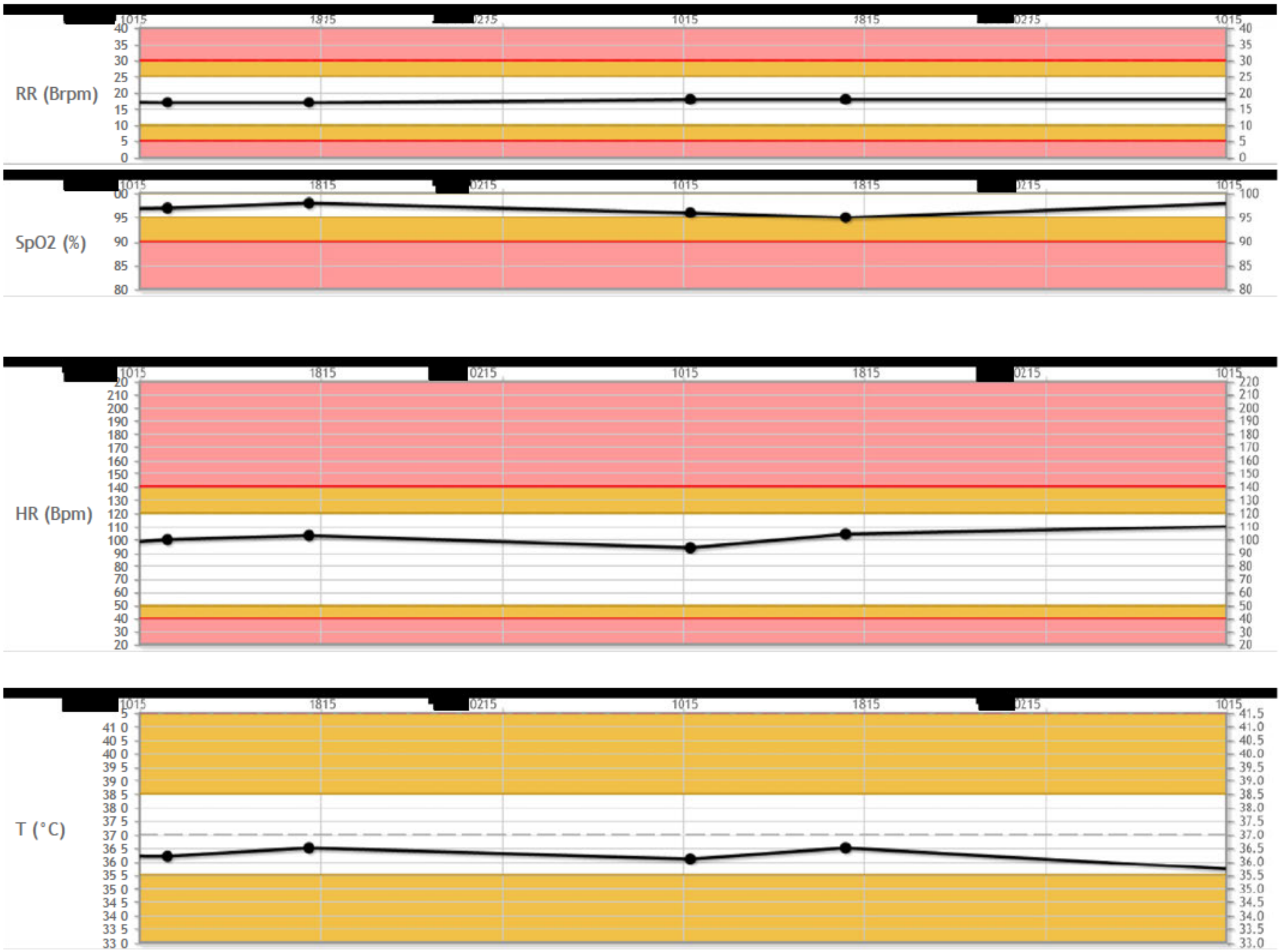

For exact values, refer to printed Medical Record or Results Flowsheet in the EMR

Last refreshed on [redacted]

Printout excludes Vital Signs with no results in this period.

[redacted]
