# Supplementary material for: Virtual Health Care for Community Management of Patients With COVID-19 in Australia: Observational Cohort Study
Source: J Med Internet Res. 2021 Mar 9;23(3):e21064. doi: 10.2196/21064 (PMC7945978; doi:10.2196/21064)
Supplement: Multimedia Appendix 3 [file jmir_v23i3e21064_app3.pdf]

Determining level of risk for rpavirtual COVID-19 patients

Patient care is tailored to individual risk. During the initial phone call, follow the flowchart using the patient's information. Once level of risk is determined, see associated box for care package.

NOTE: DAYS ARE CALCULATED BASED ON THE DATE THAT SYMPTOMS COMMENCED (DAY 0)
IF PATIENT IS AYSMPTOMATIC, COUNT FROM POSITIVE SWAB DATE (DAY 0)

Patient circumstances
Discuss with NUM if the patient is under 16 years, pregnant, or has any other factor that may require consideration.

There may be circumstances for high risk COVID-19 patients where presentations to ED may not be appropriate, such as people with palliative care needs, end stage chronic disease or complex social circumstances. In these situations, the patient must be discussed with senior medical and nursing staff in conjunction with patient's existing specialist on a case by case basis.

Inpatients
If patient is being discharged from inpatient hospital care the referring team will advise rpavirtual as to level of risk (medium or high). rpavirtual will register and deliver relevant care package

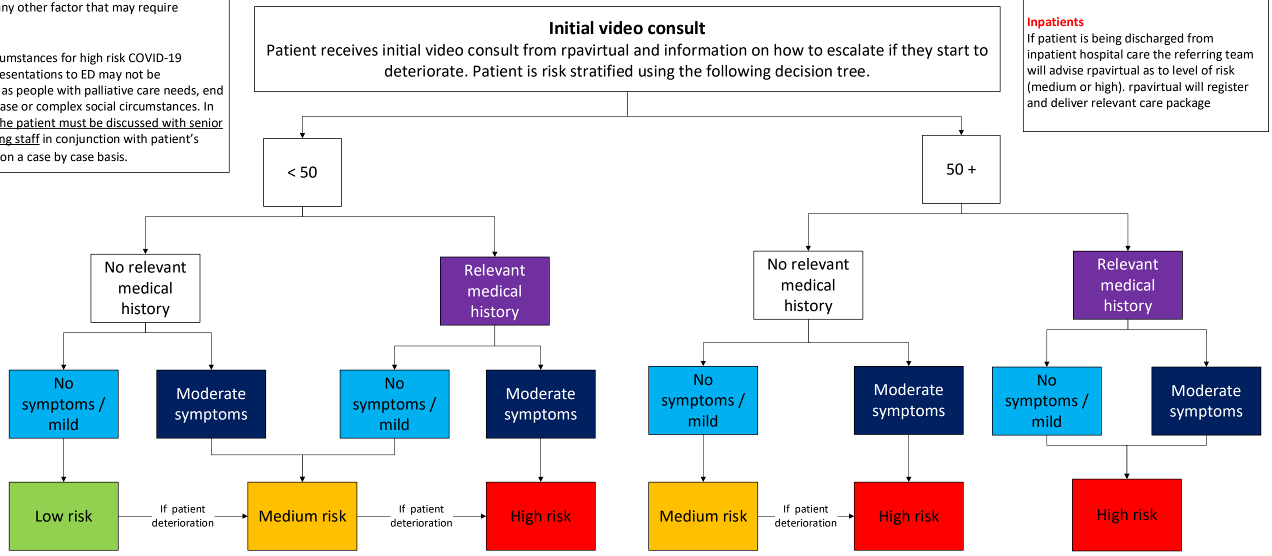

The next video consult should be scheduled based on where the patient is on the trajectory of the virus.
Example: if a patient is referred to rpavirtual on 3 July, but their symptoms commenced on 1 July, then the patient is on day 2.
Therefore the patient's next contact should be at day 3 for low and medium risk and the next day for high risk.

Low risk

- Day 3 – patient is contacted via videoconference for clinical assessment

If at day 3 patient shows signs of deterioration not requiring ED, re-categorise as medium risk and provide DAILY videoconference. Consider need for remote monitoring

- Day 10 – patient receives video call from rpavirtual for consideration of discharge
- Patient is contacted every 3 days until ready for discharge

Medium risk

- Day 3 – patient is contacted via videoconference for clinical assessment
- Day 6 to 10 – patient is contacted daily via videoconference for clinical assessment
- After day 10, patient is contacted every 3 days until ready for discharge

If at any time patient is becoming more unwell, re-categorise as high risk

High risk

- Initial consult – Wearable devices are delivered to patient home
- **Three times per day** – video call from rpavirtual which includes patient reported observations and clinical assessment.
- Patient is contacted three times per day until ready for discharge
- Consider need for increased support based on carer / nurse availability

Note: high risk COVID-19 patients in SHA should be offered wearable devices for remote monitoring. If patient declines, they are to receive in-person nursing observations from SHA nursing.

Relevant medical history

Patient has one or more of the following comorbidities:

- Under 65 years – lung disease, cardiovascular disease or renal disease (level 5 renal failure)
- 65 years and over – cancer, cardiovascular disease, hypertension, diabetes, heart failure, immunosuppression, stroke, liver disease, renal disease or lung disease

Asymptomatic / mild symptoms

Patient has:

- No symptoms
- Low grade fever < 38
- Mild cough and upper respiratory tract symptoms
- No breathlessness

Moderate symptoms

Patient has:

- Fever > 38
- Marked cough / sputum
- No or mild, stable breathlessness

Undetected deterioration including hypoxia

The rpavirtual COVID-19 protocol has been designed to detect clinical deterioration, including hypoxia irrespective of symptom severity. However, given the novel nature of the coronavirus and the related pandemic, there remains potential for undetected deterioration. It is imperative that patients understand the need for self-referral and escalation between assessments especially for low and medium risk patients not receiving wearables for home monitoring.
